# A nonfunctioning parathyroid carcinoma misdiagnosed as a follicular thyroid nodule

**DOI:** 10.1186/s12957-015-0672-9

**Published:** 2015-09-08

**Authors:** Filomena Cetani, Gianluca Frustaci, Liborio Torregrossa, Silvia Magno, Fulvio Basolo, Alberto Campomori, Paolo Miccoli, Claudio Marcocci

**Affiliations:** Endocrine Unit 2, University Hospital of Pisa, Via Paradisa 2, 56124 Pisa, Italy; Department of Surgical, Medical and Molecular Pathology and Critical Area, University of Pisa, Pisa, Italy; Department of Clinical and Experimental Medicine, University of Pisa, Pisa, Italy

**Keywords:** Primary hyperparathyroidism, Calcium metabolism, Chromogranin A, PTH, Parathyroid tumorigenesis

## Abstract

Parathyroid carcinoma (PC) is a rare endocrine malignancy. The tumor is mostly functioning, causing severe primary hyperparathyroidism, with high serum calcium and parathyroid hormone (PTH) levels. Nonfunctioning PC is extremely rare. We report a 50-year-old male patient who was referred to our Department for a right thyroid nodule, incidentally detected on carotid Doppler ultrasound scan, with a fine-needle aspiration cytology showing a follicular lesion. At the time of our evaluation, neck ultrasound showed a 1.3 cm right hypoechoic thyroid nodule with irregular margins and the absence of enlarged bilateral cervical lymph nodes. Thyroid function tests were normal. Serum calcium was normal and plasma PTH slightly above the upper limit of the normal range. The patients underwent right lobectomy. The intraoperative frozen-section pathological examination raised the suspicion of a PC. Definitive histology showed a markedly irregular infiltrative growth of the tumor with invasion of the thyroid tissue and cervical soft tissues. Immunostaining for thyroglobulin was negative, whereas staining for chromogranin A and PTH showed a strong reactivity. Based on the microscopic findings and the immunohistochemical profile, the tumor was diagnosed as a PC. Postoperative serum calcium and phosphate levels were in the normal range. One month after surgery, serum calcium and PTH were normal. Neck ultrasound and total body computed tomography scan were negative for local and metastatic disease. Eight months later, serum calcium was normal and plasma PTH level remained around the upper limit of normal range. Neck ultrasound did not show any pathological lesions. This is the first case of a nonfunctioning sporadic PC misdiagnosed prior of surgery as a follicular thyroid nodule. The parathyroid nature of the neck lesion could not be suspected before surgery. Fine-needle aspiration cytology (FNAC) may fail to distinguish a parathyroid tumor from a benign thyroid nodule because at FNAC, parathyroid and thyroid lesions have some morphological similarities. Histological criteria are not always sufficient for the differential diagnosis, which can definitely be established using immunohistochemistry.

## Background

Parathyroid carcinoma (PC) is a rare disease accounting for less than <1 % of cases of primary hyperparathyroidism (PHPT) [1, 2]. PC is usually a sporadic disease, but it has also been reported in familial PHPT, namely the hyperparathyroidism-jaw tumor syndrome (HPT-JT) and, very rarely, in the multiple endocrine neoplasia type 1 (MEN1) [3]. At variance with the asymptomatic benign counterpart, patients with PC usually present markedly elevated serum calcium and parathyroid hormone (PTH) levels [4, 2]. The clinical features are mostly due to the effects of the excessive secretion of PTH by the functioning tumor rather than to the spread of tumor mass. The management of PC is primarily surgical, with en bloc resection of the tumor with involved adjacent structures [5, 4]. Rarely, parathyroid PC has been described in patients with normal levels of serum calcium and PTH (nonfunctioning PC) [6, 7] and, with the exception of one case in the setting of MEN-2A [8], all cases reported so far are sporadic [7]. The diagnosis of nonfunctioning PC has always been established at histology. Indeed, in the absence of hypercalcemia, the parathyroid origin of the neck lump cannot be established, and the lesion is usually diagnosed as thyroid or thymic carcinoma, because of locally advanced disease (palpable neck mass, dysphagia, hoarseness due to laryngeal nerve palsy).

The suspicion of the parathyroid origin of the excised tumor may raised by the finding at histology of feature suggestive of PC, namely uniform sheets of cells arranged in a lobular pattern separated by dense fibrous trabeculae, mitotic figures within tumor parenchymal, full thickness capsular invasion with growth into adjacent tissues, and extratumoral vascular invasion [9, 10]. Nevertheless, immunohistochemical studies for PTH, thyroglobulin, thyroid transcription factor 1, and calcitonin are usually performed to establish the definitive diagnosis.

Tumor resection is rarely curative and patients mostly die for the local invasion.

## Case presentation

Written informed consent was obtained from the patient for the publication of the case report and any accompanying images.

**Fig. 1:**
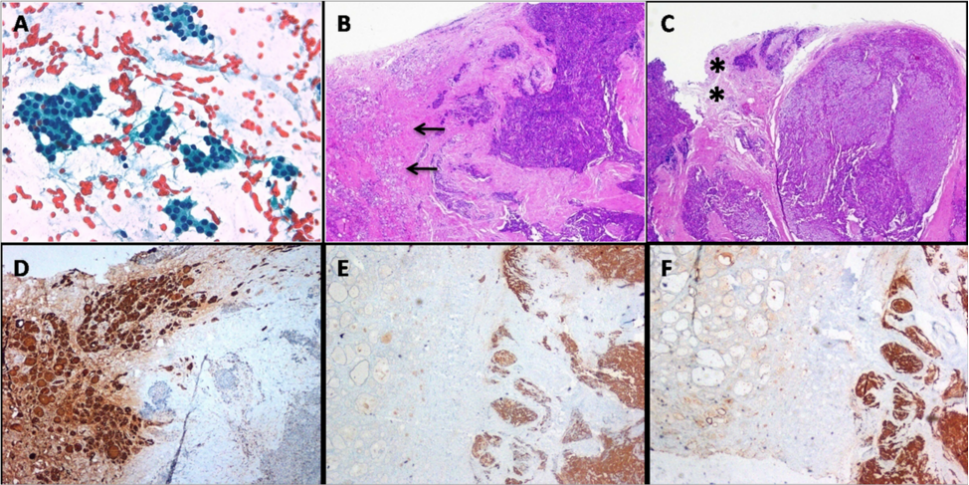
Cytological and histological specimens of nonfunctioning parathyroid carcinoma. **a** Cytological specimen showing epithelial cells organized in small cohesive clusters resembling thyroid microfollicles (Papanicolaou-stain, ×40 original magnification). **b** Histological section showing the neoplastic invasion of the neighboring thyroid tissue (*arrows* indicate the thyroid tissue; hematoxylin- and eosin-stain, ×2 original magnification). **c** Histological section showing the neoplastic invasion of the soft tissue surrounding the parathyroid tumor (*indicates the soft tissue; hematoxylin- and eosin-stain, ×2 original magnification). **d**–**f** Immunohistochemical stainings showing absence of immunoreactivity of the parathyroid tumor to thyroglobulin ((**d**), ×4 original magnification) and strong immunoreactivity to Chromogranin A ((**e**), ×4 original magnification) and parathyroid hormone (**f**), ×4 original magnification)

A 50-year-old male patient was admitted to our Department for a thyroid nodule on the right side of the neck, which was incidentally detected on carotid Doppler ultrasound scan. Fine-needle aspiration cytology (FNAC) showed a follicular lesion. At the time of our evaluation, the patient was in good health. The medical history revealed hypertension, vitiligo, and celiac disease. On physical examination, a small nodule (1 cm) was palpable in the right thyroid lobe. No enlarged neck lymph nodes were palpable. Neck ultrasound showed a 1.3 cm hypoechoic nodule with irregular margins in the right thyroid lobe together with bilateral small thyroid nodules (4–5 mm) and the absence of enlarged cervical bilateral lymph nodes. Thyroid function tests were normal with the absence of thyroid autoantibodies. Serum calcium was normal (9.7 mg/dl; normal range, 8.4–10.4 mg/dL) and PTH, routinely measured together with serum calcium in our Center in patients undergoing thyroid surgery, slightly elevated (68 pg/mL (intact PTH, 2nd generation assay; normal range, 10–65 pg/mL)). The re-review of the original slides of FNAC confirmed a follicular lesion. In particular, the cytology of the nodule showed epithelial cells with hyperchromatic nuclei organized in small cohesive clusters resembling microfollicles typically observed in thyroid follicular lesions were evident (Fig. 1a).

The patients underwent right lobectomy. During neck exploration, there were no macroscopic signs of local invasion. The intraoperative frozen-section pathological examination raised the suspicion of a PC. Definitive histology showed a markedly irregular infiltrative growth of the tumor with invasion of the thyroid tissue and cervical soft tissues (Fig. 1b, c). Immunostaining for thyroglobulin was negative, whereas staining for chromogranin A and PTH showed a strong reactivity (Fig. 1d–f). Based on the light microscopic findings and the immunohistochemical profile, the tumor was diagnosed as a PC. Postoperative serum calcium (8.7 mg/dl) and phosphate (3 mg/dl) levels were in the normal range. One month after surgery, serum calcium and plasma PTH were 9.6 mg/dL and 47 pg/mL, respectively. Neck ultrasound and total body computed tomography scan were negative for local and metastatic disease. Eight months later, serum calcium and plasma PTH levels were 9.1–9.2 mg/dl and 38–44 pg/ml (1–84 PTH 3rd generation assay, normal range, 8–40 pg/mL), respectively. Neck ultrasound did not show any pathological lesions. In order to exclude a familiar form of PHPT, in which PC may rarely occur as a nonfunctioning tumor [11], the screening of serum calcium and neck ultrasound in the first-degree relatives was normal.

### Discussion

We describe a patient with a thyroid nodule discovered at carotid ultrasound, which underwent a routine diagnostic workout, which showed typical ultrasound features and cytological evidence of a follicular thyroid lesion. Surprisingly, the histological diagnosis revealed a PC.

In experienced hand, neck ultrasound has a high sensitivity in recognizing a eutopic parathyroid lesion, but it may fail when it has an ectopic location or is located within the thyroid [12, 13]. The latter condition occurred in the patient described herein as well as in the patient described by Kim et al., who reported a 53-year-old woman with a benign thyroid nodule at neck ultrasound, initially treated elsewhere with radiofrequency ablation, which was subsequently found to be a parathyroid adenoma at surgery [14].

FNAC may also fail to distinguish a parathyroid tumor from a benign thyroid nodule because at FNAC parathyroid and thyroid lesions have some morphological similarities [15]. Indeed, colloids and macrophages, which generally are found in a thyroid nodule, can be also be present in a parathyroid lesion. On the hand, parathyroid cells are generally smaller than thyroid cells and have less cytoplasm and more chromatin, but these are not a specific signs [16].

At surgery, the intraoperative frozen-section pathology in our patient suggested that the excised lesion was a PC and not a thyroid lesion. Despite this unexpected finding, the surgical procedure was limited to the right lobectomy because the surgeon could not find any macroscopic infiltration and/or enlarged lymph nodes. The diagnosis of a PC was confirmed by definitive histology and immunohistochemistry.

To our knowledge, this is the first case of a nonfunctioning sporadic PC misdiagnosed prior of surgery as a follicular thyroid nodule. The parathyroid nature of the neck lesion could not be suspected before surgery. Indeed, serum calcium concentration was normal and PTH slightly above the upper limit of the normal range. In interpreting this latter finding, we should keep in mind that the upper normal limit is usually fixed at the 97.5 percentile of the distribution of PTH among normal individuals; therefore, some normal individuals may have slightly elevated PTH. Another explanation could be that the slight increase of PTH level could be due to a low vitamin D status, which is a rather common condition [17]. Other secondary causes of elevation of PTH, namely thiazides diuretic use and reduced renal function, were excluded. The finding that plasma PTH remained around the upper limit of normal range following parathyroidectomy supports the concept that the parathyroid tumor was nonfunctioning.

The immunohistochemical finding of PTH protein in the tumor confirms its parathyroid origin as well as its ability to transcribe the PTH mRNA. The question of whether an active PTH molecule is synthesized but not secreted or whether the PTH molecule detected at immunostaining is not the mature form of PTH, which even if secreted cannot be measured in the serum, cannot be established. Unfortunately, we do not have fresh-frozen tissue for mRNA analysis nor preoperative serum/plasma samples to further investigate these possibilities. The patient described by Baba et al. may help to shed light on this matter [18]. These authors reported a patient with classical hypercalcemic PC, in whom 2 years after parathyroidectomy, a large supraclavicular mass was detected. The patient had normal serum calcium and PTH. The lesion was excised and a recurrent, apparently nonfunctioning PC was diagnosed at histology. The patient developed transient hypocalcemia after surgery. PTH mRNA was detected in the tumor. The absence of hypercalcemia suggested that the recurrence was nonfunctioning. The authors concluded that the identification of PTH mRNA in the tumor as well as the transient hypocalcemia after surgery suggest that PTH was synthesized and secreted, albeit in amounts too small to cause hypercalcemia, but sufficient to cause suppression of the presumably normal parathyroid glands [18]. At variance with this case, our patient did not show postoperative hypocalcemia, further supporting that the tumor did not secrete full-length PTH.

Nonfunctioning PC is diagnosed in most patients in the sixth or seventh decade (age range 27–71 years) [7, 19]. The tumor size is variable but ranges between 5 and 11 cm, and in almost half of the cases, loco regional spread into thyroid, cervical soft tissues and superior mediastinum is usually present at diagnosis [7, 19]. At variance, in our patient, the diagnosis was done at an earlier age (50 years) and the tumor size was 13 mm, likely because the patient was submitted to surgery for a follicular thyroid lesion.

The histological diagnosis of PC is currently restricted to lesions showing unequivocal extra-parathyroidal growth, as evidenced by perineural invasion, full thickness capsular invasion with growth into adjacent tissues, extratumoral vascular invasion, or metastasis. The histological criteria were all present in the tumor of our patient [9]. Several immunohistochemical markers have been evaluated to further improve the diagnostic accuracy of a nonfunctioning PC [20, 21]. Positive staining of chromogranin A and synaptophysin and negative staining of thyroglobulin has been reported in nonfunctioning PC [21, 20, 22]. In our case, a positive staining for PTH and chromogranin A, and negative staining for thyroglobulin, confirmed the parathyroid nature of the tumor. Therefore, the accurate diagnosis of nonfunctioning PC should be made by considering the combination of clinical presentations, tissue morphologic structure, and immunohistological staining.

Limited information is available on the follow-up of patients with nonfunctioning PC. Recurrences occurred in about half of the patients (7), but the majority (about 80 %) was alive when reported in the literature, even though the follow-up was rather short; only four patients were followed for more than 5 years after the initial surgery [7, 19]. Long-term follow-up data and information on mortality are not available, but the advance stage of the PC at diagnosis likely predict a poor prognosis, worse than that of patients with classical hypercalcemic PC, in whom the disease extension is more limited at time of diagnosis (metastases to lymph nodes (<5 %) or distant sites (<2 %)) [2, 23]. Even though during the follow-up, the tumor usually spreads to cervical nodes (30 %) and lung (40 %) and less frequently to liver and bone (10 %); the overall survival is of about 72 and 50 % at 5 and 10 years, respectively [23, 6].

## Conclusions

This is the first case of a nonfunctioning sporadic PC misdiagnosed prior of surgery as a follicular thyroid nodule. The parathyroid nature of the neck lesion could not be suspected before surgery. FNAC may fail to distinguish a parathyroid tumor from a benign thyroid nodule because at FNAC, parathyroid and thyroid lesions have some morphological similarities. Histological criteria are not always sufficient for the differential diagnosis, which can definitely be established using immunohistochemistry. The reported case may likely contribute to enlarge the expanding clinical spectrum of nonfunctioning PC.

## Consent

Written informed consent was obtained from the patient for publication of this case report and any accompanying images. A copy of the written consent is available for review by the Editor of this journal.

## Abbreviations

FNAC: fine-needle aspiration cytology
HPT-JT: hyperparathyroidism-jaw tumor syndrome
MEN1: multiple endocrine neoplasia type 1
PC: parathyroid carcinoma
PHPT: primary hyperparathyroidism
PTH: parathyroid hormone
